# The limitations of qPCR telomere length measurement in diagnosing dyskeratosis congenita

**DOI:** 10.1002/mgg3.220

**Published:** 2016-03-20

**Authors:** Shahinaz M. Gadalla, Payal P. Khincha, Hormuzd A. Katki, Neelam Giri, Jason Y. Y. Wong, Stephen Spellman, Jack A. Yanovski, Joan C. Han, Immaculata De Vivo, Blanche P. Alter, Sharon A. Savage

**Affiliations:** ^1^ Clinical Genetics BranchDivision of Cancer Epidemiology and GeneticsNational Cancer Institute, National Institutes of Health9609 Medical Center DriveRockvilleMaryland20850; ^2^ Biostatistics BranchDivision of Cancer Epidemiology and GeneticsNational Cancer InstituteNational Institute of Health9609 Medical Center DriveRockvilleMaryland20850; ^3^ Occupational and Environmental Epidemiology BranchDivision of Cancer Epidemiology and GeneticsNational Cancer InstituteNational Institutes of Health9609 Medical Center DriveRockvilleMaryland20850; ^4^ Center for International Blood and Marrow Transplant Research500 5th St NMinneapolisMaryland55401; ^5^ Section on Growth and ObesityDivision of Translational MedicineEunice Kennedy Shriver National Institute of Child Health and Human DevelopmentNational Institutes of Health10 Center Drive, Building 10‐CRCBethesdaMaryland20892; ^6^ Departments of Pediatrics and PhysiologyUniversity of Tennessee Health Science Center and Children's Foundation Research InstituteLe Bonheur Children's Hospital50 North Dunlap Street, Room 454RMemphisTennessee38103; ^7^ Channing Division of Network MedicineDepartment of MedicineBrigham and Women's Hospital and Harvard Medical SchoolBostonMassachusetts02115; ^8^ Program in Genetic Epidemiology and Statistical GeneticsHarvard School of Public HealthBostonMassachusetts02115

**Keywords:** Bone marrow failure, diagnosis, dyskeratosis congenita, qPCR, telomere

## Abstract

**Background:**

Telomere length <1st percentile‐for‐age in leukocyte subsets by flow cytometry with fluorescence in situ hybridization (flow FISH) is highly sensitive and specific in diagnosing patients with dyskeratosis congenita (DC), a telomere biology disorder.

**Methods:**

We evaluated the clinical utility of the high‐throughput quantitative real‐time PCR (qPCR) relative telomere length (RTL) measurement as a diagnostic test for DC in patients with a priori clinical and/or genetic DC diagnoses. We calculated the sensitivity and specificity of RTL at different age‐specific percentile cutoffs in 31 patients with DC and 51 mutation‐negative relatives, and evaluated RTL difference by disease genotype.

**Results:**

qPCR RTL <1st percentile‐for‐age failed to identify more than 60% of the patients already known to have DC (sensitivity = 39%, specificity = 98%). Three‐quarters of DC patients had RTL below the 10th percentile‐for‐age (sensitivity = 74%), as did 12% of the unaffected relatives (specificity = 88%).

**Conclusions:**

Our findings suggest that the qPCR RTL method is not optimal for diagnosing DC. In light of these limitations, leukocyte flow FISH telomere length remains the recommended molecular test for diagnosing DC.

## Introduction

Dyskeratosis congenita (DC) is an inherited bone marrow failure and cancer susceptibility syndrome caused by germline mutations in telomere biology genes (Ballew and Savage 2013; Dokal et al. 2015). It is clinically diagnosed by the presence of the diagnostic triad of reticular skin pigmentation, nail dysplasia, and oral leukoplakia; however, phenotypic heterogeneity is common. Germline mutations in *DKC1* (OMIM 300126), *TINF2* (OMIM 604319), *TERC* (OMIM 602322), *TERT* (OMIM 187270), *NOP10* (OMIM 606471), *NHP2* (OMIM 606470)*, WRAP53* (OMIM 612661)*, CTC1* (OMIM 613129), *RTEL1* (OMIM 608833), *ACD* (OMIM 609377) (Kocak et al. 2014), and *PARN* (OMIM 604212) (Dhanraj et al. 2015 *;* Moon et al. 2015) genes account for approximately 70% of classic DC (Dokal et al. 2015; Bertuch 2015). Currently, telomere length (TL) less than the 1st percentile‐for‐age, measured by a standardized and validated protocol of automated multicolor flow fluorescence in situ hybridization (flow FISH) in leukocyte subsets(Baerlocher and Lansdorp 2004), has proven to be highly sensitive and specific in diagnosing DC (Alter et al. 2007, 2012). Despite its high‐performance characteristics, flow FISH TL measurement is limited because it is relatively low throughput, expensive, requires viable cells, and rapid specimen processing, and is only performed in a few clinically certified laboratories around the world. Quantitative PCR (qPCR), on the other hand, is an inexpensive method for measuring relative telomere length (RTL). This high‐throughput approach can be performed on DNA extracted from stored samples. We evaluated the clinical utility of RTL as a diagnostic tool for DC, and tested its ability to differentiate DC patients by genotype.

## Material and Method

### Ethical compliance

All participants of this study or their guardians provided written informed consent in accordance with Health and Human Services regulation 45 CFR 46.

### Study participants

This study included 31 DC patients and 51 unaffected mutation‐negative relatives who were enrolled in the NCI's IRB‐approved longitudinal Inherited Bone Marrow Failure syndromes study (NCI Protocol 02‐C‐0052, NCT‐00027274). Patients with DC included in this study had to have a germline mutation in one of the known DC‐causative genes and/or at least two features of the diagnostic triad (oral leukoplakia, reticular skin pigmentation, and/or nail dysplasia) along with other clinical findings consistent with DC (Ballew and Savage 2013; Bertuch 2015). Relatives of clinically diagnosed DC patients with unknown genetic cause were excluded from the study to avoid misclassification.

DC patients were identified as having severe marrow failure if: (1) hemoglobin <8 g/dL, absolute neutrophil count <500 cells/mcL, or platelet count <30,000/mcL, and/or (2) receiving regular blood product transfusions or androgens to treat marrow failure (Shimamura 2008).

Controls for RTL‐age standardization were derived from 477 age‐matched healthy individuals recruited for National Institute of Child Health and Development Institutional Review Board‐approved protocols *(* www.clinicalTrials.gov ID: NCT‐00320177, NCT‐00631644, NCT‐00001195 & NCT‐00001522*; n* = 104; age range 6–18 years), and from healthy donors of hematopoietic stem cells from the Center for International Blood and Marrow Transplant Research IRB‐approved Research Repository (*n* = 373; age range = 20–60)

### qPCR Telomere length measurement

QIAamp DNA Blood Maxi Kit (*n* = 373) (QIAGEN Inc, Valencia, CA), or proteinase K treatment and ammonium acetate procedure (*n* = 104) (Lofstrand Laboratories Ltd, Gaithersburg, MD) were used to extract genomic DNA from whole blood samples of the controls. Puregene (*n* = 29), Autopure (*n* = 29), or organic (*n* = 8) procedures were used for blood samples from study participants (DC patients and relatives). Information on the DNA extraction method for 16 study participants was not available. DNA volume was quantified using PicoGreen and normalized to 50 ng/*μ*L. DNA purity, represented by A260/A280 ratio for DC patients and relatives in this study ranged from 1.86 to 2.1.

Relative telomere length in peripheral blood DNA of all study participants and controls was measured using monoplex qPCR as previously described (Cawthon 2002; Wong et al. 2014). Briefly, telomere copy number was compared with a single‐copy gene (beta‐globin gene) copy number and presented as a ratio (T/S), and the final measurements were corrected for a reference sample. The reference sample contained pooled buffy coat genomic DNA with inter‐ and intra‐plate %CV's for both the telomere and single‐copy gene reactions of <0.75%. The detection ranges for telomeric and single gene reactions were 14–27 and 16–29, respectively. All samples were measured in triplicate, and 96% of them fell within the reaction detection range. The mean coefficient of variation in all samples was 0.6% for the telomere assay, 0.4% for the single‐copy gene assay, and 13.2% for the T/S measure from quality control samples. Laboratory personnel were blinded to the sample status (control, DC patients, or relative).

### Statistical analysis

Relative telomere length was log‐transformed to approximate a normal distribution (*P*‐Shapiro–Wilk test for normality = 0.85 for control, 0.25 for relatives, and 0.09 for DC patients). For control samples, we used a linear regression model to calculate the relationship between RTL and age. Our data showed the expected age‐TL inverse correlation (*r* = −0.35, *P* < 0.0001). We age‐standardized qPCR RTL in DC patients and relatives by calculating RTL percentile‐for‐age using reference percentile curves from the controls.

A Receiver Operating Curve (ROC) was constructed to test the performance of RTL in diagnosing patients with DC. The sensitivity and specificity for several percentile cut‐ off points of qPCR age‐standardized RTL were calculated. RTL in DC patients and mutation‐negative relatives, and in DC patients by genotype in two categories (*TINF2* or *DKC1* vs. other genes) were compared using Mann–Whitney test.

## Results

### Participant characteristics and qPCR RTL

DC patients were younger than their mutation‐negative relatives (median age = 21.2 years, range = 3.0–47.7 vs. 36.3 years, range = 3.8–59.4, *P*‐Mann–Whitney <0.001), and more likely to be male (74.2% vs. 45.1%, *P*‐Chi square = 0.01). Half of the patients had at least two features of the clinical triad, 42% had severe marrow failure, and all but two patients had a germline mutation identified. As expected, patients with DC had significantly shorter RTL than their mutation‐negative relatives (median RTL percentile‐for‐age = 2.7 vs. 51.2, respectively, *P* ‐Mann–Whitney <0.0001, Table 1). Our data suggested that RTL in DC patients with mutations in *TINF2* or *DKC1* (median RTL percentile‐for‐age = 0.7, range = 0.03–72) trended toward shorter RTL than DC patients with mutations in other genes (median RTL percentile‐for‐age = 4.5, range = 0.03–81.6), although not statistically significant (*P*‐Mann–Whitney = 0.07).

**Table 1:** Characteristics of study participants

	Dyskeratosis congenita (*N* = 31)	Mutation‐free relatives (*N* = 51)
Age in years; median (range)	21.2 (3.0–47.7)	36.3 (3.8–59.4)
Gender Male: Female	23:8	23:28
Genetic cause number (%)		
*TINF2*	9 (29.0%)	Mutation‐negative
*DKC1*	7 (22.6%)	
*TERC*	5 (16.1%)	
*TERT*	5 (16.1%)	
*WRAP53*	2 (6.5%)	
*RTEL1*	1 (3.2%)	
Unknown gene	2 (6.5%)	
All three features of triad	11 (35.5%)	N/A
At least two features of triad number (%)	16 (52%)	N/A
Severe bone marrow failure number (%)		N/A
Present	13 (42%)	
Absent	18 (58%)	
RTL percentile‐for‐age; median (range)	2.7 (0.005–81.3)	51.2 (0.16–99.4)

N/A , Not applicable; RTL; relative telomere length.

### Diagnostic performance of qPCR RTL

**Figure 1:**
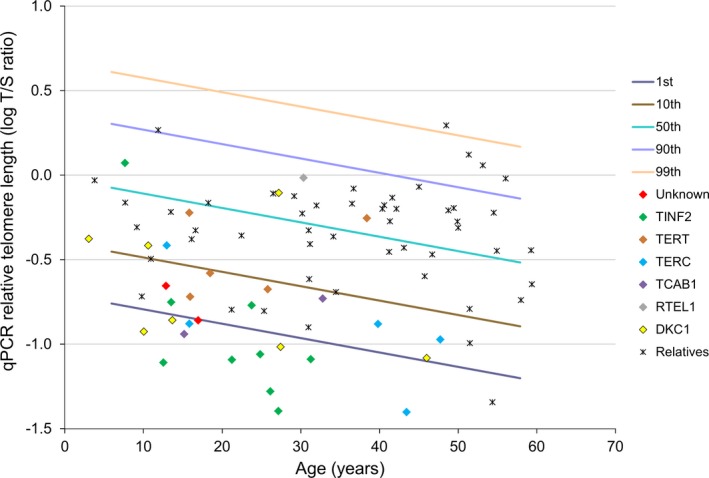
Diagnostic performance of qPCR relative telomere length in patients with dyskeratosis congenita. qPCR relative telomere length distribution of DC patients and mutation‐negative relatives in percentiles of age‐matched controls; The lines in the figures indicate the 1st, 10th, 50th, 90th, and 99th percentiles of log qPCR relative telomere length from 477 normal controls.

Figure 1 shows the distribution of RTL for DC patients by affected gene and their relatives in relation to control percentile curves. Very short RTL, defined as less than the 1st percentile‐for‐age based on qPCR RTL in controls, correctly identified less than 40% of the DC patients (sensitivity = 39%), but a high specificity (98%) was observed. RTL less than the 10th percentile‐for‐age showed improved DC diagnostic sensitivity, but much higher rate of false positives than was observed for lower cut‐off points (sensitivity = 74%, and specificity = 88%).

## Discussion

The correct diagnosis of DC is essential for proper clinical management, since treatment options, genetic counseling decisions, hematopoietic cell transplant regimens, and follow‐up strategies are significantly different for patients with inherited telomere biology disorders than those with other forms of bone marrow failure (Collins and Dokal 2015; Gadalla et al. 2013). We evaluated whether RTL measurement by the high‐throughput and less costly qPCR in peripheral blood leukocyte DNA could be an effective diagnostic method for DC. Our study showed that qPCR RTL has a low sensitivity to detect DC patients (sensitivity = 39%, and 74% at <1st, and <10th percentile‐for‐age cutoffs, respectively).

The ability of qPCR RTL to detect short telomeres has been tested in comparison with Southern blots in a previous study including patients with aplastic anemia and idiopathic pulmonary fibrosis (Gutierrez‐Rodrigues et al. 2014). The authors showed that qPCR and Southern blots agreed in identifying patients with TL <1st percentile. However, the sensitivity and specificity dropped to 40% and 63%, respectively, when patients with TL <10th percentile were considered. A direct comparison of that study with ours is limited by the unavailability of genotype data in the referenced study. Other differences that can affect study comparability include DNA extraction methods and TL presentation (log‐transformed T/S in our study vs. converted T/S to kilobases using Southern blot and qPCR data from an independent cohort in referenced study). Our data suggest that DC patients with *DKC1* and *TINF2* mutations have shorter qPCR RTL than those with mutations in other genes. These results are in agreement with previous observations of flow FISH TL in a larger sample from the same cohort. Flow FISH TL Z‐score for patients with mutations in *TINF2*,* DKC1*, or unknown genes were ‐5 versus ‐4 for the other genes, *P* = 0.01(Alter et al. 2012).

Differing proportions of leukocyte subtypes within each sample could possibly explain the limited sensitivity of qPCR RTL in correctly identifying DC patients, since qPCR provides an average RTL measure across all white blood cells in whole blood. Flow FISH TL measurement of a large cohort of healthy individuals suggested that TL differs by leukocyte subtype; specifically, TL was longer in granulocytes than lymphocytes (Aubert et al. 2012). This hypothesis was not supported by our data; in analysis comparing RTL in a subset of patients with DC (*n* = 15) who had blood count testing at the day of RTL sample collection, we found no statistical significant difference in log‐transformed RTL by granulocyte proportions below and above the median (median RTL percentile‐for‐age = 2 vs. 3, respectively, *P*‐Mann–Whitney = 0.6) (data not shown). Similarly, flow FISH data in individuals carrying *TERT* or *TERC* mutation found comparable telomere lengths in all leukocyte cell subtypes with the exception of naïve T cells and NK differentiated cells in *TERC* and *TERT* deficient individuals, respectively (Aubert et al. 2012).

Strengths of this study include clear and detailed clinical and molecular classification of DC patients and relatives. Study limitations include its relatively small sample size, a typical challenge when studying rare diseases. Our cohort may not be representative of all DC patients, particularly those with mild disease, due to possible referral bias. Additionally, we used several DNA extraction methods in our study, which might have affected the results. It has been suggested that RTL measured from QIAamp^™^‐extracted DNA are shorter than those of DNA extracted by PureGene^™^ or phenol/chloroform methods; this observation was attributed to differences in DNA purity (Cunningham et al. 2013). However, the DNA used in our study had little protein contamination based on A260/A280 ratios >1.8.

In conclusion, qPCR RTL less than the 1st percentile‐for‐age had low sensitivity for identifying patients with DC. Raising the cutoff to the 10th percentile improved the sensitivity at the expense of poor assay specificity. Our findings suggest that qPCR RTL is not optimal for diagnosing DC.

## Conflicts of Interest

None declared.
